# Magnetic Resonance Imaging-Guided Stereotactic Body Radiation Therapy for Medically Inoperable Endometrial Cancer

**DOI:** 10.7759/cureus.35215

**Published:** 2023-02-20

**Authors:** Neris Dincer, Ufuk M Abacioglu, Evrim Tezcanli, Gorkem Gungor, Meriç Şengöz

**Affiliations:** 1 Radiation Oncology, Acibadem Mehmet Ali Aydinlar University School of Medicine, Istanbul, TUR; 2 Radiation Oncology, Acibadem Altunizade Hospital, Istanbul, TUR; 3 Radiation Oncology, Acibadem Maslak Hospital, Istanbul, TUR

**Keywords:** magnetic resonance imaging-guided online adaptive radiotherapy, gynecological cancer, mr-guided stereotactic body radiotherapy, geriatric oncology, endometrial cancer

## Abstract

Endometrial carcinoma is the most frequently diagnosed gynecological cancer among women aged 50 and older in developed countries. In patients who are not amenable to surgery, radiotherapy results in improved survival with acceptable adverse effect profiles. Definitive stereotactic body radiotherapy (SBRT) as a monotherapy remains an unaddressed concept in the literature. Here, we present the case of an 86-year-old woman who was diagnosed with early-stage endometrial carcinoma and was medically inoperable due to cardiac comorbidities. She was treated with magnetic resonance imaging-guided online adaptive radiotherapy-based SBRT. She tolerated the treatment well, with mild increased vaginal discharge. Complete metabolic and radiological responses were obtained. She continues to be disease free in the first year of treatment with no long-term side effects. Our protocol presents promising results with a safe toxicity profile for inoperable early-stage endometrial cancer. Future studies are warranted in light of the current knowledge.

## Introduction

Endometrial carcinoma is the most frequently diagnosed gynecological cancer among women aged 50 and older in developed countries [1]. Most cases present in the early stage, and surgery is the first-line treatment with staging mainly based on surgical findings. Radiotherapy (RT) and chemotherapy are indicated in the adjuvant setting for patients with additional high-risk features, including histological type, grade, International Federation of Gynaecology and Obstetrics (FIGO) stage, depth of invasion, and lymphovascular invasion [2].

In patients who are not amenable to surgery, RT results in improved survival with acceptable adverse effect profiles. Most patients are treated with external-beam radiotherapy (EBRT) followed by brachytherapy or brachytherapy alone [3]. Patients who are not a candidate for brachytherapy because of comorbidities can be selected for conventional fractionation EBRT-alone [4] or a boost can be delivered with stereotactic body radiotherapy (SBRT) [5]. Definitive SBRT for inoperable patients as a monotherapy remains an unaddressed concept in the literature.

Herein, we report a case of medically inoperable endometrial carcinoma treated with magnetic resonance imaging-guided online adaptive radiotherapy (MRgRT)-based SBRT.

## Case presentation

**Figure 1:**
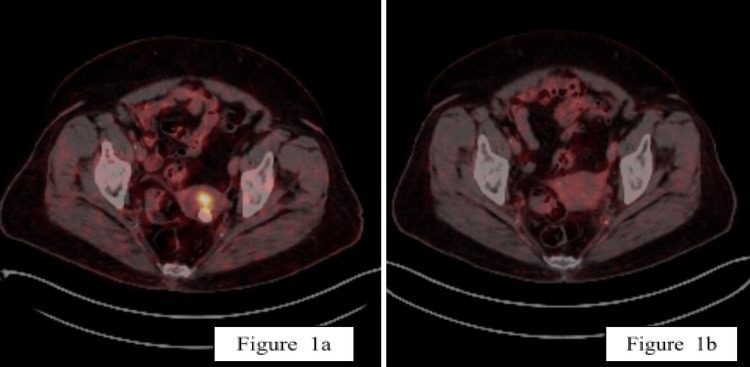
PET-CT scans prior to treatment and after treatment. (a) PET-CT scan before treatment. (b) PET-CT scan three months after treatment showing complete metabolic response. PET-CT = positron emission tomography-computed tomography

The patient was an 86-year-old woman with comorbidities due to compensated heart failure, hypertension, and type 2 diabetes mellitus being treated with oral medications and subcutaneous insulin. The patient had dyspnea with mild physical effort. She had no surgical or family history of malignancy. Her last menstruation was at the age of 50. She presented to the gynecology department with a complaint of postmenopausal bleeding that occurred twice during the month before her initial diagnosis. Transvaginal ultrasonography findings were consistent with a heterogeneous uterus and an irregular endometrium with a thickness of 8 mm without any visible adnexal pathology bilaterally. An endometrial biopsy revealed a grade 2 endometrioid carcinoma. Positron emission tomography-computed tomography (PET-CT) did not show any evidence of regional or distant metastasis and confirmed the primary disease with a maximum standardized uptake (SUVmax) value of 16.5 and a calcified 17 mm focus in the uterus (Figure 1, Panel a). She was clinically staged as FIGO stage I because the disease was found to be confined to the corpus uteri.

She was deemed medically inoperable due to cardiac comorbidities. She denied conventional RT due to the extensive number of daily hospital visits. She was found to be at high risk for any procedure that required anesthesia, and she denied invasive procedures under local anesthesia; therefore, brachytherapy only was not an option for this patient. The patient opted for a non-invasive SBRT treatment course with MRgRT and informed consent was obtained.

**Figure 2:**
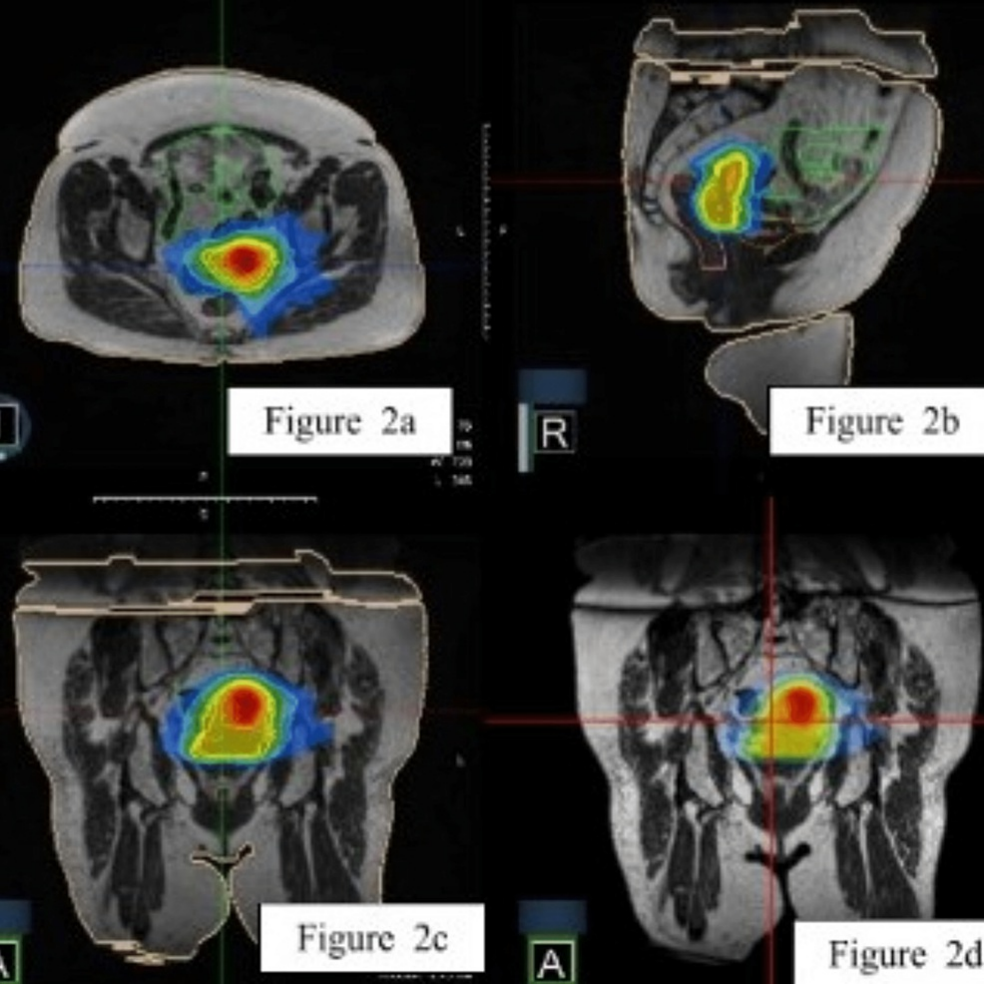
Treatment plan. (a) Isodose color-wash view in the axial plane. (b) Isodose color-wash view in the sagittal plane. (c-d) Isodose color-wash view in the coronal plane. Shades of red demonstrate 100-105%, orange-yellow areas demonstrate 80-70%, greenish shades show 50-60%, and bluish shades show 30-40% isodose line.

**Figure 3:**
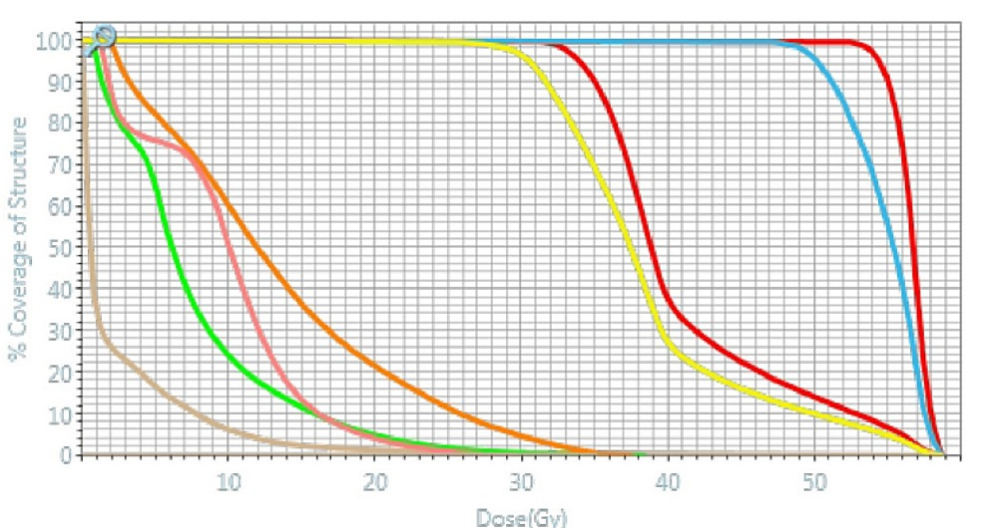
Dose-volume histogram of the reference plan. Red line on the right: GTV; red line on the left: CTV; blue line: PTV50Gy; yellow line: PTV30Gy; orange line: bladder; pink line: rectum; green line: bowels; beige line: skin CTV = clinical target volume; GTV = gross tumor volume; PTV = planning target volume

Before the simulation and each treatment, the patient was asked to empty her bladder and drink 300 mL water with a 30-minute wait before the treatment to achieve a bladder volume consistency of 250-300 cc. She was recommended to start a special diet to reduce constipation and gas a few days before the simulation. As per our standard clinical protocol, enemas were planned if the patient presented with a full rectum that might cause interfraction variation and dosimetric changes. Simulation CT and planning 0.35 Tesla MRI images on MRIdian® Linac (ViewRay Inc Mountain View, CA) were acquired. MRI scans were fused with PET-CT and the gross tumor volume (GTV) was delineated as the visible tumor. The uterus and cervix were defined as the clinical target volume (CTV). Margins of 3 mm were added to CTV to form the planning target volume (PTV) receiving 30 Gy and to GTV to form PTV50Gy. Organs at risk (OARs) were determined as the rectum, bladder, sigmoid colon, small bowel, and femoral heads. A step-and-shoot intensity-modulated radiotherapy (IMRT) treatment plan with a simultaneous integrated boost technique was produced using 27 fields. Departmental dose constraints detailed in Table 1 were applied. This reference plan consisted of 27 beams of 6 MV flattening filter-free photons with a single isocenter. A total dose of 50 Gy in five fractions (10 Gy/fraction) was prescribed to PTV50Gy and a total dose of 30 Gy in five fractions (6 Gy/fraction) was prescribed to the PTV30Gy with biologically effective dose values to the target of 100 Gy and 48 Gy, respectively. Treatments were delivered every other day. Volumes of PTV50Gy and PTV30Gy were 11.62 cc and 135.77 cc, respectively. The max point dose was 58.8 Gy. Contours and isodose color-wash views in axial, sagittal, and coronal views are presented in Figure 2, and the dose-volume histogram of the reference plan is shown in Figure 3.

**Table 1:** Departmental dose constraints. OARs = organs at risk; Gy = Gray

OARs	Constraint
Rectum	<0.10 cc at 38.06 Gy
	<1.00 cc at 36.25 Gy
	<5.00 cc at 34.43 Gy
	<10.00 cc at 32.62 Gy
	<20.00 cc at 25.00 Gy
	<35.00% at 18.00 Gy
Bladder	<0.10 cc at 38.06 Gy
	<1.00 cc at 36.25 Gy
	<15.00 cc at 32.62 Gy
	<43.00% at 17.50 Gy
Bowels	≤0.50 cc at 36.00 Gy
	≤1.00 cc at 33.00 Gy

A three-dimensional MRI dataset was obtained during each fraction, and the anatomy of the day was evaluated by a radiation oncologist. GTV, CTV, and OARs were recontoured accordingly. An online adaptive plan was produced and re-optimized for all fractions mostly to improve target volume coverage. The CTV was selected as the target volume to be constantly monitored in a single sagittal plane with an image rate of four frames per second using a 3 mm boundary margin for auto-beam on and off gating. The patient’s on-couch total time was 35 minutes (range: 32-45 minutes).

The patient tolerated the treatment well with grade I mild vaginal discharge as per the Common Terminology Criteria for Adverse Events version 5.0 during the course of treatment without any other accompanying symptoms. She was initially evaluated for treatment response with an MRI scan two months after the completion of treatment, which revealed complete regression of the uterine mass. Her follow-up PET-CT scan obtained three months after the treatment revealed a complete metabolic and anatomic response (Figure 1, Panel b). As per our institutional follow-up protocol, she was being followed up with pelvic MRI scans every three months without recurrence or symptoms one year after treatment.

## Discussion

Endometrial carcinoma is the most common gynecological malignancy in developed countries, with most cases confined to the uterus at the time of diagnosis [6]. Tumor grade is a prognostic factor reported to be associated with disease-free and overall survival in endometrial carcinoma patients [6]. The mainstay of treatment is deemed to be a total hysterectomy with bilateral salpingo-oophorectomy. Lymph node dissection is also recommended to determine the stage of the patient and tailor the adjuvant treatment approach accordingly [2]. Although the FIGO staging system is based on surgical findings, MRI and ultrasonography can aid in determining the extent of invasion [7,8] and PET is reported to be sensitive and specific for demonstrating nodal metastases [9].

Surgery alone followed by routine examinations is accepted to be sufficient for low-risk patients but adjuvant RT and/or chemotherapy is indicated based on several criteria such as histological type, grade, FIGO stage, depth of invasion, and lymphovascular invasion [10].

In medically unfit patients, following the clinical staging, EBRT is an alternative for both curative and palliative intent and offers acceptable local control and safety profile as a monotherapy or in combination with brachytherapy depending on the individual risk factors [11,12]. Brachytherapy may not be applicable for fragile patient groups because these patients are not amenable to anesthesia for the same reasons as they cannot undergo surgery [13]. This patient group can be treated with EBRT alone with an external boost to the gross disease [14]. A systematic review of 2,694 endometrial cancer patients who were treated with RT alone (EBRT+BT: 47.7%; BT alone: 51.3%; EBRT alone: 1.2%) reported that combined disease-specific survival for all groups was 78.5%. Grade 3 or more toxicities were low for all groups (3.7% for EBRT+BT; 1.8% for BT alone, and 1.2% for EBRT alone) [12].

Conventionally fractionated EBRT has an ongoing role in both adjuvant and definitive treatment of endometrial carcinoma. However, the role of SBRT is not well established. There are studies that use SBRT for a boost dose following pelvic radiotherapy [14,15]. Jones et al. evaluated the dosimetric feasibility of SBRT in the definitive setting in medically inoperable endometrial cancer patients who were previously treated with high-dose-rate brachytherapy [16]. CTVs were delineated as uterus plus cervix, and a dose of 34 Gy in four fractions was prescribed to the target. They generated helical tomotherapy plans and compared them to prior intracavitary brachytherapy plans. They reported superior target coverage with SBRT compared with intracavitary brachytherapy. OAR doses were higher with SBRT, although not outside tolerance limits. Kemmerer et al. treated 11 stage I-III endometrium cancer patients who were not amenable to brachytherapy with EBRT (4,500 cGy; 180 cGy/fraction) followed by an SBRT boost with a prescribed dose of 3,000 cGy in five fractions. No severe acute or late toxicity was observed, and they reported that this technique was feasible [5].

In SBRT, normal organ protection is as crucial as target coverage because high ablative doses are prescribed to the lesion. The endometrium is in close proximity to the bladder and intestines and these structures are subject to daily anatomic variations. The internal motion of pelvic organs is also worth mentioning. Daily cone-beam CT with or without fiducial marker implementation is used in conventionally fractionated schemes [17] but surrogates introduce a level of uncertainty and risk in SBRT. A retrospective study evaluating the daily shifts, systematic errors, and margin requirements in endometrial cancers treated with IMRT concluded that a margin of 7-10 mm is the minimum margin required to avoid any uncertainty but that this margin needs to be further expanded in patients with a high body mass index [18]. An accurate target volume delineation is the first step toward accurate target coverage and appropriate critical organ preservation and CT alone has insufficient soft-tissue discrimination. There are studies combining CT with ultrasound and MRI for target delineation that report how uterine segmentation and the extent of disease improve when other imaging modalities complement CT [19].

Integration of an MRI and a linear accelerator led us to take advantage of soft-tissue discrimination of MRI as well as online, real-time visualization of the target that resulted in precise delivery of the prescribed dose [20]. Our patient was medically inoperable and could not receive brachytherapy for the same reason, and she denied any long-term treatment. SBRT was the treatment of choice and an ablative dose was delivered in a short fractionation regimen. Portelance et al. used MRgRT for the sequential boost following EBRT on the conventional linear accelerator of a patient with vaginal vault recurrence. They prescribed 28 Gy in four fractions to the boost, and the conventional scheme was 45 Gy in 25 fractions [21]. We prescribed 50 Gy in five fractions to the primary tumor to yield a similar biologically equivalent dose. Online adaptive planning with daily contouring enabled us to safely deliver the dose with tighter margins than mentioned in the literature [18]. Our patient did not suffer from any serious side effects during our limited follow-up. Her MRI scan two months after treatment showed complete regression of the uterine mass, and PET-CT three months after treatment showed a complete metabolic response indicating that the treatment was a success.

There are also several reports on the use of MRgRT for the treatment of other gynecological malignancies. Hes et al. reported their preliminary experience with MRgRT for cervical cancer patients ineligible for brachytherapy boost and suggested that higher doses could be safely delivered to the target lesions when compared to cone-beam CT-based treatments [22].

Hadi et al. investigated the feasibility and safety of an MRg-SBRT boost following EBRT for 10 patients with locally advanced cervical cancer and recurrent vaginal and cervical tumors [23]. They concluded the feasibility of the treatment when brachytherapy normal tissue constraints were followed. However, long-term follow-up as well as higher patient numbers are needed to validate the results of these case series.

## Conclusions

Herein, we present a case of definitive SBRT to an endometrium cancer case with MRgRT guidance. Our protocol presents promising results for inoperable early-stage patients and supports minimum side effects and an early good response. Future studies are warranted in light of the current knowledge.
